# Determinants of Resistance to Checkpoint Inhibitors

**DOI:** 10.3390/ijms21051594

**Published:** 2020-02-26

**Authors:** Linda Tran, Dan Theodorescu

**Affiliations:** 1 Department of Surgery (Urology), Cedars-Sinai Medical Center, Los Angeles, CA 90048, USA; Linda.Tran2@cshs.org; 2 Cedars-Sinai Samuel Oschin Comprehensive Cancer Institute, Los Angeles, CA 90048, USA; 3 Department of Pathology and Laboratory Medicine, Cedars-Sinai Medical Center, Los Angeles, CA 90048, USA; 4 Cedars-Sinai Health System, 8700 Beverly Blvd., OCC Mezz C2002, Los Angeles, CA 90048, USA

**Keywords:** cancer, immunotherapy, combination therapy, immune checkpoints, immune checkpoint inhibitors, functional genomics, next generation sequencing, prognostic markers, predictive markers

## Abstract

The development of immune checkpoint inhibitors (ICIs) has drastically altered the landscape of cancer treatment. Since approval of the first ICI for the treatment of advanced melanoma in 2011, several therapeutic agents have been Food and Drug Administration (FDA)-approved for multiple cancers, and hundreds of clinical trials are currently ongoing. These antibodies disrupt T-cell inhibitory pathways established by tumor cells and thus re-activate the host’s antitumor immune response. While successful in many cancers, several types remain relatively refractory to treatment or patients develop early recurrence. Hence, there is a great need to further elucidate mechanisms of resistant disease and determine novel, effective, and tolerable combination therapies to enhance efficacy of ICIs.

## 1. Introduction

Because of their effectiveness, several immune checkpoint inhibitors (ICIs) are Food and Drug Administration (FDA)-approved for a wide range of cancer types [1]. Ipilimumab was the first ICI to be approved in 2011 for the treatment of advanced melanoma. This monoclonal antibody targets cytotoxic T-lymphocyte-associated protein 4 (CTLA-4), an inhibitory checkpoint on activated T cells. Similarly, monoclonal antibodies targeting the programmed cell death protein 1/programmed death-ligand 1 (PD-1/PD-L1) axis exploit a negative co-stimulatory signal meant to mitigate T-cell receptor (TCR) signaling. Blockade of these inhibitory signals reactivates the host antitumor immune response with consequent destruction of the tumor. The development of ICIs has drastically altered the landscape of cancer treatment and are especially appealing due to possible long-term durable responses. However, current therapies are not successful in all patients; even in generally responsive tumors and in certain tumor types, most patients do not respond or acquire early resistance. To increase efficacy, ICIs are being tested in combination with each other as well as other therapies targeting T-cell activation or pathways in the tumor microenvironment. In this review, we outline currently understood mechanisms of immune checkpoint inhibitor resistance and discuss the approaches used for discovery of rational combinations that can overcome such resistance.

## 2. Immune Checkpoint Inhibitors

### 2.1. CTLA-4

The human immune system has evolved pattern recognition receptors over a millennium to protect the host from foreign pathogens and developed mechanisms to screen immune cells from recognizing self-molecules. Adaptive cell-mediated immune responses are carried out by T cells, which are a type of lymphocyte that becomes activated to specific antigens to either directly (through cytotoxic CD8+ T cells) or indirectly (through helper CD4+ T cells) destroy abnormal cells. Overall, T helper (Th) cells regulate the activity of other immune cells by releasing T-cell cytokines. They are also important for B cell antibody class switching, activation, and growth of cytotoxic T cells, and in maximizing bactericidal activity of phagocytes, such as macrophages [2]. The cytokine secretion patterns of Th cells can further categorize these effector cells as type 1 (Th1) or type 2 (Th2), with type 1 producing interferon gamma (IFN-γ) and type 2 producing interleukin-4 (IL-4), IL-5, and IL-13 [3]. The activation of T cells requires engagement of a co-stimulatory molecule on a professional antigen presenting cell (APC) in addition to the TCR. Physiologically, this secondary signal prevents recognition of self-peptides, and the absence of such a signal results in T-cell anergy. Naïve T cells constitutively express CD28, the receptor for CD80 (B7-1) and CD86 (B7-2) proteins. Interaction of T-cell CD28 with B7-1 or B7-2 on APCs facilitates maturation, proliferation, activation, and survival of the former. Upon activation, T cells induce expression of CTLA-4 that attenuates signaling by competitively binding to B7-1 and B7-2 [4,5], with higher affinity than CD28; thus, inhibiting further activation and leading to immunological tolerance and prevention of autoimmunity. Following TCR signaling, additional CTLA-4 is recruited to the immunologic synapse via intracellular vesicles to spatially regulate inhibition [6]. CTLA-4 can also deplete available ligand from neighboring cells through trans-endocytosis. For example, B7-1 and B7-2 are degraded in CTLA-4+ cells, resulting in reduced co-stimulation through CD28 [7]. Immunotherapy using CTLA-4 blockade aims to sterically hinder interactions with B7 ligands [8] and thereby facilitate continued positive co-stimulation with CD28. This promotes antitumor response through expansion of tumor-infiltrating T cells and possibly the depletion of regulatory T-cell (Treg) populations, which are immunosuppressive and downregulate the activation and proliferation of effector T cells [9].

### 2.2. PD-1/PD-L1

PD-1 is a cell surface receptor expressed on activated T and B lymphocytes to regulate peripheral tolerance and T-cell responses. Binding of the receptor to its ligands PD-L1 or PD-L2 results in apoptosis of antigen-specific T cells, reduced TCR-mediated activation, attenuation of T-cell proliferation, and reduced apoptosis of Tregs, culminating in the inhibition of T-cell activity. These ligands are expressed on APCs and non-lymphoid tissues and can be induced by inflammatory cytokines, such as IFN-γ and IL-10 [10]. PD-L1 is frequently expressed on tumor cells, or in the tumor microenvironment, and allows evasion of immune detection [11]. The cytoplasmic region of PD-1 contains two tyrosine motifs, immunoreceptor tyrosine-based inhibition motif (ITIM) and immunoreceptor tyrosine-based switch motif (ITSM), which are phosphorylated upon receptor binding. Src homology 2-domain-containing tyrosine phosphatase 2 (SHP2) and SHP1 are then recruited to these phosphorylated motifs [12,13]. In T cells, PD-1 signaling results in dephosphorylation of key TCR signaling components, most notably CD28 and the ZAP70/CD3zeta signalosome [14,15]. This interference leads to reduced cytokine production, proliferation, and effector function. PD-1 blockade releases inhibition of TCR signaling and restores effector function of exhausted CD8+ T cells. The concept of “T-cell exhaustion”, first described during viral infections, is characterized by the stepwise and progressive loss of T-cell functions, and can culminate in the physical deletion of the responding cells. In cancer, these cells can remain in the microenvironment and exhibit reduced cytotoxic activity, disrupted cytokine production, and expression of inhibitory receptors [16]. Thus, PD-1 blockade supports a more effective antitumor immune response through reactivation of effector function. However, it is important to note that only certain subsets of exhausted T cells respond to PD-1/PD-L1 blockade, independent of PD-1 expression [15].

### 2.3. Additional T Cell Inhibitory and Stimulatory Pathways

While CTLA-4 and PD-1/PD-L1 ICIs have been shown to be effective as mono- and dual-therapeutic agents, other T-cell inhibitory and stimulatory pathways are currently being investigated for synergistic effect. Markers previously utilized to identify T-cell exhaustion are now being explored as potential targets for immune cell reactivation. Lymphocyte activation gene 3 (LAG-3) is a cell surface receptor expressed on activated T cells, B cells, natural killer cells, Tregs, and dendritic cells. LAG3 has structural homology to CD4 and can bind their shared ligand, major histocompatibility complex class II (MHC class-II) molecules, on APCs with a higher affinity [17]. LAG3 maintains T-cell homeostasis and negatively regulates their expansion [18]. T-cell immunoglobulin-3 (TIM-3) is a cell surface receptor expressed on activated T cells, Tregs, and white blood cells that mediate innate (non-specific) immunity. Engagement of TIM-3 in effector Th1 cells results in an influx of calcium and apoptosis [19]. T cell immunoglobulin and immunoreceptor tyrosine-based inhibitory motif domain (TIGIT) is a surface protein expressed on T cells and natural killer (NK) cells. Binding of TIGIT to poliovirus receptor inhibits natural killer cell cytotoxicity and inhibits T-cell activation through the maturation of immunoregulatory dendritic cells [20,21]. V-domain Ig suppressor of T-cell activation (VISTA) is an immune checkpoint molecule expressed on myeloid cells, T cells, and regulatory T cells. VISTA can function as an inhibitory receptor on T cells to suppress activation or as a ligand on APCs to modulate T-cell proliferation and cytokine production. Blockade of LAG-3, TIM-3, TIGIT, and VISTA have shown improved anti-tumor responses in preclinical models when combined with anti-CTLA-4 or anti-PD-1 therapy and are currently being evaluated in clinical trials. Detailed information on trials can be found at the https://clinicaltrials.gov/ web site.

Alternatively, agonists of T-cell activation are being explored to revitalize anticancer immune response. Several members of the tumor necrosis factor receptor superfamily (TNFRSF) have been identified as potential targets due to their functions in the regulation of immune cell populations implicated in cancer progression. TNFRSF4, or OX40, is expressed on CD4+ and CD8+ T cells, NK cells, and neutrophils. Stimulation of OX40 prolongs T-cell division and prevents apoptosis, resulting in increased numbers of surviving T cells that develop into memory T cells [22]. TNFRSF18, or glucocorticoid-induced TNF receptor-related protein (GITR), has been described in multiple immune cell types, most notably Tregs, and is found highly expressed on CD8+ and CD4+ T cells following TCR activation [23,24,25]. Signaling through GITR drives a high-avidity CD8+ T-cell response to tumor antigens, increases effector T-cell proliferation via expression of cytokines IL-2 and IFN-γ, and reduces the suppressive function of Tregs [26,27,28]. TNFRSF9, also referred to as 4-1BB or CD137, is expressed on NK cells, macrophages, B cells, and activated T cells. Stimulation of 4-1BB preferentially modulates CD8+ T cells, resulting in the amplification of populations in vivo and protection from activation-induced cell death [29,30]. TNFRSF5, or CD40, is broadly expressed amongst many cell types, including immune, vascular, and epithelial cells [31]. Ligation of CD40 on APCs supports maturation, which facilitates T-cell activation and differentiation [32]. Inducible T-cell co-stimulator (ICOS) is expressed predominately on activated CD4+ cells. Upon binding to its ligand (ICOSL), ICOS stimulates T-cell proliferation and cytokine production [33]. Recent findings have identified ICOS-mediated phosphoinositide 3-kinase (PI3K) signaling as necessary for transcription factor T-box expressed in T cells (T-bet) expression during anti-CTLA-4 therapy [34]. T-bet regulates commitment to the Th1 lineage and controls IFN-γ production in CD4+ T cells and natural killer cells [35,36]. These data suggest ICOS may be a promising target to heighten Th1-mediated anti-tumor responses. TNFRSF7, or CD27, is highly expressed on the surface of activated T cells and shed into circulation as soluble CD27 [37]. CD27 stimulates the survival of activated T cells throughout the many rounds of division [38]. Agonists of these immune stimulatory pathways have demonstrated synergistic anti-tumor responses when used in conjunction with ICIs prompting clinical trials.

## 3. Mechanisms of Resistance to Immune Checkpoint Blockade

While anti-CTLA-4 and anti-PD-1/PD-L1 immune checkpoint blockade has shown varying success in cancers, including melanoma, non-small cell lung carcinoma (NSCLC), and urothelial carcinoma, many show low or no response, including gastrointestinal, breast, pancreatic, prostate, sarcoma, and colorectal cancers [39,40,41,42,43,44,45,46,47]. Resistance to immunotherapy is classified as primary resistance in which the cancer is completely refractory or acquired resistance, in which there is initial response yet the cancer relapses and progresses [48]. In this section of the review, we will cover tumor cell-intrinsic and -extrinsic factors that contribute to these forms of immune checkpoint blockade resistance.

### 3.1. Tumor Mutational Burden

Neoantigens are tumor-specific peptides that result from somatic mutations in cancer cells. It is hypothesized that the number of non-synonymous single nucleotide variants that occur in a tumor is predictive of responsiveness to immune checkpoint blockade therapy. A higher occurrence of these mutations, also referred to as the tumor mutational burden (TMB), increases the likelihood of producing immunogenic peptides that can be recognized by the host immune system. In support of this, deficiencies in DNA damage repair pathways underwrites a hyper-mutational phenotype in which cells accumulate many DNA mutations, and are enriched in tumors of patients with durable responses to anti-PD-1 therapy [40]. Notably, tumors that present microsatellite instability as a result from impaired DNA mismatch repair are especially susceptible to immune checkpoint blockade [49]. Gubin et al. showed that tumor antigen-specific T cells are present in tumors and reactivated with the application of immune checkpoint blockade. Moreover, genomics and bioinformatics were utilized to identify tumor-specific mutant proteins as a primary class of antigens that leads to T cell mediated rejection of tumors [50]. In melanoma, urothelial carcinoma, small cell lung cancer, and NSCLC, high TMB has been shown to be significantly associated with ICI response [40,41,51,52,53,54]. The prognostic value of TMB for immune checkpoint blockade relies on the assumption that there is a direct correlation between the number of neoantigens and robustness of immune infiltration. However, TMB is not predictive of immune infiltration in cancers driven by copy number alterations, such as breast, pancreas, and bladder [55]. Furthermore, even in cancers with a strong association, neoantigen load alone cannot predict ICI response outcome of individual patients. In these cancers, additional stratification based on neoantigen heterogeneity may improve survival predictions. Neoantigens can be presented on all tumor cells (clonal) or in a subpopulation of tumor cells (subclonal). Tumors with high burden of clonal neoantigens were demonstrated to respond to ICIs while poor responders were enriched for subclonal neoantigens [56].

### 3.2. Tumor Microenvironment

The tumor microenvironment (TME) is a complex milieu of cancer, stromal, and immune cells. Various T-cell subsets have been implicated in modulating response to immunotherapy [57]. Multiple studies have found that the exclusion of CD8+ T cells from the TME is correlated with poor clinical outcome [58,59,60]. The degree of immune infiltration in the TME is generally characterized into three classes: immune-desert, which lacks immune cell infiltration, immune-excluded, which has immune cells in the surrounding stroma (but not penetrating the tumor), and immune-inflamed, which has immune cells adjacent to tumor cells [61,62]. Immune-inflamed or ‘hot’ tumors generally contain immune and tumor cells expressing immune checkpoint molecules and are associated with a more favorable response to ICI therapy [63]. Additionally, a T-cell-inflamed gene signature related to IFN-γ signaling is predictive of anti-PD-1 response across multiple cancers [64]. In opposition, the recruitment of Treg cells, a subset of CD4+ T cells, inhibits anti-tumor immune responses, and is correlated with poor prognosis [65]. Tumor- and stroma-specific signaling pathways have been identified that contribute to T-cell exclusion and therefore resistance to ICI. Tumor cell-specific activation of the WNT β-catenin pathway has been correlated to the absence of T cells in metastatic melanoma and shown to result in T-cell exclusion and ICI resistance in a preclinical model [66]. It is hypothesized that in immune-excluded tumors, stroma contributes to T-cell exclusion by forming a physical extracellular matrix barrier around tumor cells [67]. This is supported by a study that showed inhibition of transforming growth factor-β (TGF-β) in fibroblasts aided T-cell penetration and improved responses to anti-PD-LI therapy in a preclinical model [68]. Furthermore, the extracellular matrix secreted by stroma can influence the migration and positioning of T cells [69]. Cancer-associated fibroblasts (CAFs), the most abundant stromal cell type in tumors, can mediate T-cell death and dysfunction through PD-L2 and FASL [70]. Factors in the TME can thus affect efficacy of ICIs [71]. 

### 3.3. Genomic Drivers

Immunoediting is a term that describes the sculpting of tumor cell immunogenicity as the tumor evolves [72]. In ICI-resistant tumors, immunoediting can lead to loss of immunogenic antigens through clonal elimination or chromosomal deletions [73]. This may lead to a tumor that lacks specific neoantigens and can evade immune detection. A crucial step in immune recognition of tumor cells is neoantigen presentation, which occurs through human leukocyte antigen (HLA) class I complexes. Neoantigens may not be presented due to loss of antigen expression or genetic deficiencies in antigen processing or presenting machinery [74,75]. Beta-2-microglobulin (B2M) is important for HLA class I folding and expression at the cell surface. Inactivation of B2M causes defects in antigen presentation, and is enriched in patients that are non-responsive to immune checkpoint blockade [76,77].

A T-cell-inflamed gene signature containing IFN-γ signaling is prognostic of patients that will derive benefit from ICIs [64]. IFN-γ is a cytokine produced by activated T cells and natural killer cells that can inhibit proliferation of targeted cells and enhance immune function through magnification of antigen presentation on APCs. Janus kinase 1 (JAK1) and JAK2 are receptor-associated kinases essential in the initiation of the IFN-γ signaling cascade. Loss-of-function mutations in JAK1 or JAK2 abrogates response to IFN-γ, desensitizing tumor cells to IFN-induced growth inhibition [78]. Furthermore, JAK1/JAK2-deficient tumor cell populations can silence HLA class I antigen presentation, which hinders T-cell recognition [79]. Additionally, cancer cell expression of PD-L1 protects against IFN cytotoxicity through inhibition of signal transducer and activator of transcription 3 (STAT3) phosphorylation, an important transcription factor in the IFN signal transduction pathway [80]. Genomic defects of genes in the IFN-γ pathway have been associated with lack of response to ICI and are implicated in primary and acquired resistance [81,82].

Aberrant signaling through mechanisms, such as mitogen-activated protein kinase (MAPK), PI3K, Wnt, and IFN can negatively affect immune cell recruitment and function; therefore, influence response to ICI therapy [83]. Loss or inactivation of tumor suppressor genes serine/threonine-protein kinase (STK11) and phosphatase and tensin homolog (PTEN) has been associated with poorer response to ICIs and reduced intratumoral T-cell populations [84,85,86]. Intratumoral immune exclusion can also result from activating oncogenic signaling in pathways, such as TGF-β or WNT/β-catenin. TGF-β signaling in the stromal compartment of the TME can induce a myofibroblastic barrier to restrict T cell infiltration, while β-catenin can reduce dendritic cell recruitment and priming of T cells [66,68]. Mutational status of epidermal growth factor receptor (EGFR) has been suggested as a predictor of anti-PD-1 resistance, due to EGFR-mutant positive lung patients demonstrating low response and deriving no overall survival benefit [87,88]. A preclinical model of EGFR-driven lung tumors has shown abnormal signaling can remodel the microenvironment through the expression of immunosuppressive factors to evade immune detection [89].

### 3.4. Host-Specific Genetic Variation

Response to immune checkpoint blockade relies on T-cell recognition of tumor epitopes to induce anti-tumor activity. The HLA complex is the set of genes encoding cell-surface proteins involved in antigen presentation and are therefore essential in immunosurveillance. Antigens presented on HLA class I are recognized by CD8+ T cells, while antigens presented on HLA class II are recognized by CD4+ T cells. For class I molecules, these peptides are stably bound within a binding pocket consisting of six pockets comprised of polymorphic amino acid residues that establish binding avidity and functionality [90,91,92]. Thousands of functional variants have been observed in the genes, which encode HLA class I molecules, HLA-A, -B, and -C [93]. These variants confer binding of select repertoires of peptides, referred to as the human immunopeptidome. Heterozygosity of HLA-I loci is predicted to result in presentation of a more diverse collection of tumor neoantigens and therefore improved response to ICI [94,95]. Analysis of patients with advanced melanoma or NSCLC treated with immune checkpoint blockade demonstrated homozygosity in at least one HLA-I loci, is associated with reduced survival. Interestingly, this correlation remains significant in the context of mutation load, tumor stage, and drug class [96]. Furthermore, patients with HLA-1 heterozygous loci exhibited increased clonal expansion of TCR repertoires, possibly conferring a stronger T-cell response [96]. In patients, loss of heterozygosity in HLA loci, mutation of HLA genes, and modulation of HLA expression may reduce response to immune checkpoint blockade.

The gut microbiome has been implicated in mediating antitumor immune action and response to ICI. Fecal microbiome samples of patients whom responded to anti-PD-1 treatment presented increased alpha diversity and abundance of *Ruminococcaceae* bacteria. This profile was associated with stronger antitumor immunity in patients and was recapitulated in a germ-free mouse model [97]. Antibiotic treatment of patients with advanced NSCLC, renal cell carcinoma, or urothelial carcinoma whom received anti-PD-1 therapy is correlated with shorter progression-free survival and overall survival [98]. Quantitative metagenomics of patients before and after anti-PD-1 treatment revealed a higher richness in the composition of the gut microbiota with improved clinical response. In these patients, enrichment of the commensal *Akkermansia muciniphila* was most associated with responders to immune checkpoint blockade [98].

Disruption of the microbiota can modulate myeloid-derived cell responses in the tumor microenvironment and dampen response to immunotherapy and chemotherapy [99]. These myeloid cells originate from monocytes and granulocytes and are stimulated by tumor-derived factors to remain in activated immature states that may be tumor-promoting. Included in this classification are myeloid-derived suppressor cells (MDSCs), which are defined by their ability to suppress T cells and tumor-associated macrophages (TAMs) [100]. Furthermore, mice fed with *Bifidobacterium* demonstrated reduced tumor growth and greater intratumoral numbers of CD8+ T cells. Notably, *Bifidobacterium* administration displayed synergistic anti-tumor responses with anti-PD-L1 therapy [101]. These studies illustrate the influence of the gut microbiota on immune cell function and highlight dysbiosis as in important field in the context of immune checkpoint blockade therapy.

## 4. Combinations with Immune Checkpoint Inhibitors

Monotherapy ICIs have durable response rates in subsets of patients in many, but not all, cancer types. To extend the efficacy of ICIs to all patients and cancer types, studies exploring synergistic activity with conventional therapies, immune therapies, and small molecule inhibitors are being performed. In addition to offering improved clinical outcomes, these treatments may also offer a more tolerable safety profile for patients with less drug-related adverse events.

### 4.1. Anti-CTLA-4 and Anti-PD-1

Perhaps unsurprisingly, the combination of anti-CTLA-4 and anti-PD-1 treatments resulted in longer overall survival in patients with advanced melanoma, renal-cell carcinoma, and DNA mismatch repair-deficient/microsatellite instability-high metastatic colorectal cancer [102,103,104]. Though both therapies target immune checkpoints that attenuate T-cell activation, they do so through distinct mechanisms that differentially affect specific T-cell populations [105]. Anti-PD-1 monotherapy results in the expansion of exhausted CD8+ T cells, while dual therapy results in the expansion of activated terminally differentiated effector CD8+ T cells [106]. Anti-CTLA-4 monotherapy increases the expansion of Th1-like CD4+ T cells, while dual therapy further increases the frequency of this population [106,107]. These data confirm that combinational therapies benefit from unique mechanisms of action that cannot be inferred from monotherapies alone. Clinical trials for anti-CTLA-4 and anti-PD-1 combinational therapy have demonstrated promising anti-tumor activity in lung cancers, mesothelioma, esophagogastric cancer, prostate cancer, and sarcoma [108,109,110,111,112,113].

### 4.2. Chemotherapy, Radiotherapy, and Surgery

Chemotherapy and radiotherapy can sensitize tumor cells to ICIs by increasing immunogenicity following cellular death. The release of tumor antigens and danger-associated molecular patterns (DAMPs) may positively affect immune cell recognition of aberrant cells and prime an efficient immune response [114,115]. This process is referred to as immunogenic cell death (ICD) and is characterized by the translocation of calreticulin (CRT) to the cell surface and release of adenosine triphosphate (ATP) and high mobility group box 1 (HMGB1). Anthracyclines, oxaliplatin, and mafosfamide are able to induce ICD through the production of reactive oxygen species (ROS) and endoplasmic reticulum (ER) stress [116]. Conversely, chemotherapeutics such as cisplatin and mitomycin C are weak inducers of ER stress and do not trigger translocation of CRT and subsequent ICD [117,118]. Additionally, immunosuppressive cells, such as Tregs and MDSCs, are diminished from the TME following treatment, facilitating the infiltration of cytotoxic T cells [119,120,121]. In patients with metastatic NSCLC, improved progression-free survival and overall survival has been observed with the addition of immune checkpoint blockade therapy to chemotherapy [122]. A preclinical model of mesothelioma demonstrated that concomitant treatment with anti-CTLA-4 and gemcitabine resulted in synergistic anti-tumor effect, while phased administration resulted in no significant difference as compared to gemcitabine alone [123]. Clinical data from triple negative breast cancer patients support a short-term induction period of doxorubicin or cisplatin increases the likelihood of response to anti-PD-1, and enriches immune-related genes, including T-cell cytotoxicity and JAK-STAT pathways [124]. Similarly, a phase 2 international study found anti-CTLA-4 treatment following paclitaxel and carboplatin improved immune-related progression-free survival, while concurrent treatment showed no improvement [125]. These studies indicate the timing of immune checkpoint inhibitor administration is key to clinical benefit, likely due to chemotherapy-induced antigen release. Furthermore, concurrent versus phased administration may vary dependent on chemotherapeutic properties and cancers, warranting additional study.

Similar to certain chemotherapeutic agents, radiotherapy has been shown to induce ICD, resulting in phagocytosis of tumor cells, processing of tumor antigens, and priming of CD8+ T cells [126]. In contrast to the pro-immunogenic effects of radiotherapy, there are also immunosuppressive effects from the killing of CD8+ T cells, preservation of Tregs, and activation of TGF-β [127,128]. It is proposed that the combination of ICIs can ameliorate these mechanisms of immunosuppression. In a preclinical model of breast cancer, ionizing radiation and anti-CTLA-4 treatment conferred survival advantage over either alone through the inhibition of lung metastases [129]. Subsequent studies have demonstrated combination with immune checkpoint blockade therapy increases survival in models of glioblastoma multiforme, hepatocellular carcinoma, melanoma, NSCLC, colorectal cancer, and other cancers [130,131,132]. Combined with immune checkpoint inhibitors, radiotherapy can induce antigen-specific immune responses. This was shown to result from increased presence of antigen-experienced T cells and memory T cells. Furthermore, radiotherapy improved antigen cross presentation in lymph nodes, increased intratumoral T cells, and enhanced presentation of tumor-specific antigens [133]. Tumor cells can also adapt to radiotherapy by upregulating expression of PD-L1. In models of melanoma, colorectal, and triple-negative breast cancer, this upregulation resulted from IFN-γ produced by CD8+ T cells. The concurrent administration of anti-PD-L1 treatment was able to increase efficacy of radiotherapy and prevent recurring tumors in long-term surviving cohorts [134]. Additionally, anti-PD-L1 reduces tumor-infiltrating MDSCs through TNF [135]. A phase 3 clinical trial administering anti-PD-L1 following a combination of chemotherapy and radiation therapy showed significantly longer progression-free survival in NSCLC patients [136]. Interestingly, radiotherapy has been observed to have a systemic effect on the reduction of tumors outside the initial treatment field, termed an abscopal effect [137]. These reports have become more frequent with the addition ICIs, with preclinical and clinical data indicating immune checkpoint blockade potentiates abscopal effects [138,139,140,141]. These studies indicate sequencing and timing of radiotherapy and ICIs are likely to affect efficacy. Arguments have been proposed for the administration of immunotherapy prior, concurrent, and subsequent to radiotherapy. Radiotherapy results in the destruction of tumor infiltrating lymphocytes (TILs), which have migrated within tumor margins and demonstrate increased immunological activity towards cancer cells [142]. Therefore, immunotherapy delivered prior to radiotherapy is exerted on increased numbers of TILs and readies the adaptive immune system to respond to antigens released by radiation-induced tumor cell death [143]. Concurrent treatment of immunotherapy and radiotherapy has demonstrated regression of brain metastases in advanced melanoma [144]. When delivered prior to immunotherapy, radiotherapy may maximize immunologic priming—facilitating a stronger immune response to a wider range of epitopes [136,145]. Combinational therapy has demonstrated synergistic anti-tumor effect; however, further study is required to determine optimal timing of treatment.

Major surgery is a common treatment for many solid cancers. While it is nearly impossible to prove the consequence of this, there is some evidence that surgery impacts the rate of systemic tumor cell dissemination into blood vasculature, release of growth factors, and immune suppression [146,147,148,149]. Thus, surgical resection may create a high-risk window during which cells can spread and the host is immunosuppressed. To combat these negative events, ICIs are being explored as neoadjuvant therapies prior to surgery [150]. In preclinical models, blockade of the PD-1/PD-L1 axis post-surgery reduces tumor recurrence and restores dysfunction of CD8+ T cells [151,152]. Moreover, blockade before surgery in the clinical setting has contributed to the expansion of neoantigen-specific T-cell clones in NSCLC patients [153]. Similarly, the administration of CTLA-4 blockade therapy has demonstrated increased activation of T-cell populations. In patients with urothelial carcinoma, treatment with anti-CTLA-4 preoperatively resulted in increased frequency of CD4 + ICOShi T cells, which was then retrospectively shown to correlate with overall survival for metastatic melanoma patients [154]. Furthermore, patients with advanced melanoma exhibited decreased circulating MDSCs and increased intratumoral CD8+ T cells following neoadjuvant anti-CTLA-4 treatment [155]. 

### 4.3. Vaccines and Adoptive Cell Therapies

Cancer vaccines utilize tumor-specific antigens to stimulate a specific antitumor immune response but have had few clinical successes. With the development of ICIs and the approval of Sipuleucel-T for the treatment of prostate cancer in 2010, vaccination has become an appealing avenue to increase TILs in poorly immunogenic tumors [156,157]. In preclinical models of poorly immunogenic melanoma and prostate cancer, the combination of anti-CTLA-4 and granulocyte/macrophage colony-stimulating factor (GM-CSF)-expressing vaccine had significant synergistic effects in eradicating tumors and eliciting an immune response dependent on increased CD8+ T cells [158,159]. Interestingly, data indicate optimal benefit is in part dependent on sequence of therapeutic administration, with cohorts receiving anti-CTLA-4 post vaccination exhibiting higher levels of CD8+ T cells and increased tumor-specific lytic function [160]. This additive effect has also been observed in pancreatic and glioma models of cancer, where it was shown that GM-CSF vaccination induced expression of PD-1/PD-L1 and facilitated the action of anti-PD-1 therapy [161,162]. In these models, combinational therapy exerted a significant survival advantage, while neither therapy alone induced benefit. In the clinical setting, anti-CTLA-4 treatment in conjunction with GM-CSF vaccination has demonstrated promising results in pancreatic adenocarcinoma. Dual treated patients demonstrated improved overall survival and enhanced T-cell response [163]. Peptide- and viral-based vaccines have also validated clinical benefit of combinational therapy in human papilloma virus (HPV)-driven cancer and advanced solid cancers [164,165].

Adoptive cell therapy (ACT) is a form of immunotherapy that has achieved durable responses in cancer patients. ACT involves the autologous infusion of TILs or modified immune cells expressing unique T-cell receptors (TCR) or chimeric antigen receptors (CAR), which recognize tumor epitopes to mediate anti-tumor responses [166]. Current protocol for TIL therapy in melanoma patients consists of isolating lymphocytes from resected tumor fragments, ex vivo expansion of populations with tumor cell-killing capacity, and a lymphodepleting preparative regimen prior to reinfusion [167]. While initially most successful in hematological malignancies, ACT has now been established as a powerful treatment option for advanced melanoma and is further being explored in other solid tumors, such as breast and NSCLC [167,168,169,170]. However, challenges to the development of efficacious therapies include lack of tumor-specific antigens, difficulty in penetrating desmoplastic stroma, and an immunosuppressive microenvironment [171,172]. Furthermore, modified lymphocytes can still become exhausted and express immune checkpoints, which hinder activity [173]. The combination of immune checkpoint blockade to ACT in a preclinical model of melanoma has shown that ICIs can aid in the reversion of immunosuppressive TMEs and confer long-term protection against recurrence of solid tumors [174]. Additionally, ICIs can reinvigorate the immune system and support the persistence of CAR T cells in preclinical and clinical models [175,176,177]. Adjuvant immune checkpoint blockade appears to enhance the potency of CAR T-cell therapy and may extend the success observed in hematologic cancers to solid cancers.

### 4.4. Tumor Microenvironment Factors

Heterogeneity of the TME may largely influence the efficacy of immune checkpoint blockade. Of note are the various populations of immunosuppressive cells, such as MDSCs, tumor-associated macrophages (TAMs), and CAFs that can modulate the immune microenvironment, enhance tumor cell growth, and support immunotherapy resistance. Increased intratumoral presence of immunosuppressive myeloid cells is correlated with poor prognosis and response to immune checkpoint blockade [178,179]. Furthermore, it has been demonstrated that TAMs can capture anti-PD-1 monoclonal antibodies via Fcγ receptors and thereby prevent binding to CD8+ T cells [180]. TAMs are educated by the TME and are broadly classified as M1 or M2 macrophages. M1 macrophages are classically activated macrophages, which produce proinflammatory cytokines and are generally considered anti-tumoral. M2 macrophages produce anti-inflammatory cytokines and inhibit CD8+ T cells. This phenotype is considered pro-tumoral [181]. While T cells are generally the focus when discussing immune checkpoint inhibitors, TAMs also express PD-1. In a preclinical model, blockade of the PD-1/PD-L1 axis enhances macrophage phagocytosis and reduces tumor growth. Furthermore, PD-1+ TAMs expressed a surface profile similar to M2 macrophages [182]. Anti-PD-1 treatment in a model of osteosarcoma pulmonary metastasis resulted in increased migration of M1 macrophages and decreased migration of M2 macrophages [183]. Therapies targeting the interactions and functionality of myeloid cells in the TME have shown promising results in refractory models of cancer. Entinostat is a class-I-selective histone deacetylase (HDAC) inhibitor that improves immune checkpoint therapy by reducing the immunosuppressive ability of MDSCs [184]. Colony-stimulating factor 1 receptor (CSF-1R) blockade results in reduced immune suppression by diminishing the number of TAMs, reprogramming residual TAMs to encourage antigen presentation, and supporting T-cell activation [185]. Focal-adhesion kinase (FAK) is a non-receptor tyrosine kinase, which mediates proliferation, survival, and migration of multiple cell types in the TME. In addition to protecting tumor cells from anoikis, FAK is important for the migration and proliferation of CAFs, and the immunosuppressive capabilities of MDSCs, TAMs, and Tregs [186,187,188]. Inhibition of FAK reduces intratumoral stroma and myeloid cells and potentiates immune checkpoint blockade therapy [189].

Inflammatory mediators secreted by cells in the TME can promote cancer development [190]. Cyclooxygenase (COX) activity has been identified as a driver of immune suppression and inhibitors have demonstrated synergistic effect with immune checkpoint blockade through the partial restoration of tumor immunosurveillance [191,192]. 

### 4.5. High-Throughput Methods of Discovering Combinational Therapies

The increasing availability of high-throughput screening technologies has permitted the rational discovery of ICI combinational treatments. Small-molecule and genome-wide libraries have been utilized to identify and target mechanisms, which mediate T-cell interactions with cancer cells. To facilitate time to U.S. FDA approval, multiple studies have highlighted promising combinational therapies through customized screens focused on previously approved medications. Lizotte et al. developed an in vitro luciferase-based screening assay to identify small molecules, which modulate T-cell-mediated killing of tumor cells and validated epidermal growth factor receptor (EGFR) inhibitors at potentiators of anti-PD-1 immunotherapy [193]. Tu et al. utilized in vivo functional genomics to identify discoidin domain receptor 2 (DDR2) as a target mechanism to sensitize multiple tumor types to anti-PD-1 treatment [194]. In more encompassing studies, genome-wide CRISPR-Cas9 screens have been leveraged to further elucidate genes, which permit tumor cell escape from immunosurveillance. Patel et al. utilized an in vitro co-culture method to assemble a comprehensive set of genes whose loss in tumor cells resulted in reduced function of effector CD8+ T cells [195]. Importantly, this study linked their gene set to loss-of-function mutations in immunotherapy refractory patients, thereby proposing novel targets of immune escape for combinational therapies. Recent advances in technology allow the screening of curated CRISPR-Cas9 libraries in innate and adaptive immune populations in vivo to better delineate gene function [196]. Utilizing CHIME, an immune editing system based on chimeric bone marrow, LaFleur et al. were able to implicate the phosphatase PTPN2 in the differentiation of exhausted CD8+ T cells via modulation of type 1 IFN signaling [197]. 

## 5. Conclusions

The application of immune checkpoint inhibitors in the treatment of cancer has given us the first real glance at durable responses in patients. This has created a burgeoning field to discover combinations that extend benefits to multiple tumor types and those that are refractory. While the first wave of combinational therapies focused on mechanisms previously implicated in cancer progression, there is now a push to discover novel synergistic treatments via high-throughput screening methods. To derive the most value, there is a necessity to elucidate pathways that allow tumor cells to circumvent immune detection and identify which patients will benefit most from specific combinations.
